# Closed-Loop Control Better than Open-Loop Control of Profofol TCI Guided by BIS: A Randomized, Controlled, Multicenter Clinical Trial to Evaluate the CONCERT-CL Closed-Loop System

**DOI:** 10.1371/journal.pone.0123862

**Published:** 2015-04-17

**Authors:** Yu Liu, Min Li, Dong Yang, Xuena Zhang, Anshi Wu, Shanglong Yao, Zhanggang Xue, Yun Yue

**Affiliations:** 1 Department of Anesthesiology, Beijing Chaoyang Hospital, Capital Medical University, Beijing, People’s Republic of China; 2 Department of Anesthesiology, Zhongshan Hospital, Fudan University, Shanghai, People’s Republic of China; 3 Department of Anesthesiology, Union Hospital, Tongji Medical College, Huazhong University of Science and Technology, Wuhan, People’s Republic of China; Erasmus Medical Centre, NETHERLANDS

## Abstract

**Background:**

The CONCERT-CL closed-loop infusion system designed by VERYARK Technology Co., Ltd. (Guangxi, China) is an innovation using TCI combined with closed-loop controlled intravenous anesthesia under the guide of BIS. In this study we performed a randomized, controlled, multicenter study to compare closed-loop control and open-loop control of propofol by using the CONCERT-CL closed-loop infusion system.

**Methods:**

180 surgical patients from three medical centers undergone TCI intravenous anesthesia with propofol and remifentanil were randomly assigned to propofol closed-loop group and propofol opened-loop groups. Primary outcome was global score (GS, GS = (MDAPE+Wobble)/% of time of bispectral index (BIS) 40-60). Secondary outcomes were doses of the anesthetics and emergence time from anesthesia, such as, time to tracheal extubation.

**Results:**

There were 89 and 86 patients in the closed-loop and opened-loop groups, respectively. GS in the closed-loop groups (22.21±8.50) were lower than that in the opened-loop group (27.19±15.26) (p=0.009). The higher proportion of time of BIS between 40 and 60 was also observed in the closed-loop group (84.11±9.50%), while that was 79.92±13.17% in the opened-loop group, (p=0.016). No significant differences in propofol dose and time of tracheal extubation were observed. The frequency of propofol regulation in the closed-loop group (31.55±9.46 times/hr) was obverse higher than that in the opened-loop group (6.84±6.21 times/hr) (p=0.000).

**Conclusion:**

The CONCERT-CL closed-loop infusion system can automatically regulate the TCI of propofol, maintain the BIS value in an adequate range and reduce the workload of anesthesiologists better than open-loop system.

**Trial Registration:**

ChiCTR ChiCTR-OOR-14005551

## Introduction

With the development of fast- and short-acting anesthetics, such as propofol and remifentanil, total intravenous anesthesia (TIVA) has been widely accepted because it is fast-acting and stable, and allows for a rapid recovery. Advances in target controlled infusion (TCI) further improved TIVA to better fit the pharmacokinetics and pharmacodynamics of these drugs. However, individual differences among patients impair the application of the pharmacokinetic model of TCI in some patients. For this reason, closed-loop controlled infusion of propofol has been descripted since the 1980’s [1–3]. With the development of computer technology and EEG monitoring technology, many new closed-loop systems have been invented in recent years [4–10]. Closed-loop controlled infusion of anesthetics can avoid the limitations of TCI by compensating the disturbances caused by individual differences, and thus helps to achieve a rational use of anesthetics. Closed-loop controlled infusion can also decrease the anesthesiologist’s workload [11].

Anesthetics alter the electrocortical activity in a dose-dependent manner. Bispectral index (BIS), a FDA-approved index that has been widely used to monitor the depth of anesthesia, has been used in several comparative studies involving the infusion of propofol, and can help regulating the infusion of anesthetics during general anesthesia [4,5,7–10]. So we choose BIS as the index for monitoring closed-loop controlled infusion of propofol.

The CONCERT-CL closed-loop infusion system designed by VERYARK Technology Co., Ltd. (Guangxi, China) is an innovation using TCI combined with closed-loop controlled intravenous anesthesia under the guide of BIS. Therefore, the aim of the present study was to investigate whether the CONCERT-CL system could be better to stabilize the BIS and maintain the BIS value between 40 and 60 by comparing the effects of BIS-guided regulated closed-loop TCI of propofol and manually regulated TCI of propofol.

## Materials and Methods

### Subjects

The protocol for this trial and supporting CONSORT checklist are available as supporting information; see S1 CONSORT Checklist and S1 Protocol.

The present study was approved by the ethical committees of each participating centers (Beijing Chaoyang Hospital Ethics Committee, Shanghai Zhongshan Hospital Ethics Committee and Wuhan Union Hospital Ethics Committee), and all subjects provided a written informed consent. We didn’t registered the trial before enrolment of participants started, because we thought that the trial registration was not necessary for every chinical trial. Before the submission of our manuscript we had registered the trial at ChiCTR and the trial registry number is ChiCTR-OOR-14005551. The authors confirm that all ongoing and related trials for this intervention are registered. We can ensure we report the date at which the ethics committee approved the study as well as the complete date range for patient recruitment.

From 2012-12-1 to 2013-6-30, 180 patients were included in this study (n = 60 each hospital). All the patients undergone general anesthesia and with an expected operation time>120 minutes were enrolled in three hospitals (Beijing Chaoyang Hospital, Capital Medical University, Shanghai Zhongshan Hospital, Fudan University and Wuhan Union Hospital, Tongji Medical College, Huazhong University of Science and Technology) and were randomly assigned to two groups (closed-loop group and opened-loop group) (n = 90 each). Age ranged from 18 to 65 years. ASA classification was I or II. Patients with psychiatric disorders, spinal cord diseases, with a history of cerebral surgery, or with cardiac pacemaker were excluded. Only experienced anesthesiologists were allowed to conduct TCI of propofol and remifentanil, as well as to monitor BIS.

### Study procedures

The patients were randomly assigned to the closed-loop or opened-loop group using a random number table. Group assignment was enclosed in opaque envelopes before the operation.

Blood pressure, electrocardiogram, and pulse oximetry were monitored. Neuromuscular blockade at the abductor pollicis muscle was monitored using the neuromuscular blockade monitoring system provided by VERTARK Technology Co., Ltd. (Guangxi, China), while BIS was monitored using an A-2000XP BIS (Aspect Medical systems, Dublin, Ireland).

Parameters of TIVA-TCI described by Marsh et al. [12] and Minto et al. [13] were used for propofol and remifentanil, respectively.

The induction phase was defined as from the infusion of propofol (Diprivan, AstraZeneca, London, UK) and remifentanil (Remifentanil Hydrochloride for Injection, Yichang Humanwell, Yichang, China) to a BIS maintained at <60 for 30 seconds. The maintenance phase was defined as from the end of the induction to the end of the infusion of propofol and remifentanil [14].

Midazolam (1–2 mg) was intravenously administered as a premedication. The initial target concentrations of propofol in the plasma (2 to 4 ug/ml) and remifentanil (4 to 8 ng/ml) in the induction phase were selected by the anesthesiologists according to their clinical experience. In the maintenance phase, the target concentration of propofol was adjusted manually to maintain the BIS at about 50 (40 to 60) in the opened-loop group, while the target concentration of propofol in the closed-loop group was adjusted automatically by the system. The TCI of remifentanil was used in both groups, and the target concentration (2 to 8 ng/ml) was based on clinical judgment of the anesthesiologists.

The closed-loop infusion of rocuronium (Esmeron, Merck Forsst, Montreal, Canada) was used after the induction phase. The induction dose of rocuronium was 0.6 mg/kg, and then the feedback parameter was at reappearance of second twitch (the count 2) for maintenance infusion of rocuronium. Endotracheal intubation or laryngeal mask insertion was performed when TOFr = 0. The anesthesiologists were allowed to administer the drugs manually or switch the closed-loop infusion to manual infusion of the drugs during the operation, if needed.

All aspects of anesthesia managements except for the drug infusion were performed by the anesthesiologists according to the currently used guidelines. No inhalation anesthetic was used. The infusion of the muscle relaxants was stopped at about 30 minutes before the end of the operation, and 100 to 200 mg of tramadol was administered at about 20 minutes before the end of the operation.

The infusion of propofol and remifentanil was stopped at the same time after the operation in both groups. Then, muscle-relaxant antagonists (1 mg of atropine and 2 mg of neostigmine) were administered. The endotracheal tube or laryngeal mask was removed when the patients reached consciousness, could respond to the clinicians, had a restored autonomous respiration, SpO_2_>95%, TOFr>90%, and were without hemodynamic disturbance.

Global score (GS) [14,15] could reflect the overall performances of the closed-loop infusion system, including the fluctuation of BIS, the proportion of time of adequate anesthesia (BIS between 40 and 60), median absolute performance error (MDAPE), and Wobble [16]. Therefore, GS was selected as the primary outcome. The parameters were calculated as follows:

Performance error (PE) was defined as the difference between the actual value and the set value:

$$PEij=\left(\frac{BISactualij-BISset}{BISset}\right)\times 100$$

Median performance error (MDPE):

MDPEi=Median[PEij,j=1,…,Ni]

Median absolute performance error (MDAPE):

MDAPEi=Median[|PEij|,j=1,…,Ni]

Wobble reflects the intraindividual variability in PE:

Wobblei=[|PEij−MDPEi|,j=1,…,Ni]

*i* = subject number; j = j^th^ (one) measurement of observation period; N = total number of measurements during the observation period.

Global score (GS) was calculated using the formula:

$$GS=\frac{MDAPE+Wobble}{\%of\;time\;BIS\;between\;40\;and\;60}$$

A lower GS, meaning lower MDAPE, lower Wobble, and higher proportion of time of BIS between 40 and 60, represented better performances of the closed-loop infusion system.

The secondary outcomes included the percentage of adequate anesthesia (BIS between 40 and 60), overshoot (BIS<40) and undershoot (BIS>60) periods, occurrence of suppression ratio (SR) defined as SR>10% lasting at least one minute, and parameters (PE, MDPE, MDAPE, Wobble). And the adjustment times per hour for control of adequate anesthesia (BIS between 40 and 60).

The secondary outcomes also included doses of propofol, remifentanil and rocuronium, and the endotracheal tube removal time (from the end of the infusion of propofol and remifentanil to the removal of the endotracheal tube).

PE, MDPE, MDAPE, Wobble, GS, and the proportion of the time of BIS were collected automatically by the data-collecting software provided by VERYARK Technology Co., Ltd. The trends of BIS, target concentration of the drugs, and neuromuscular blockade could be displayed.

### Statistical analysis

SPSS 19.0 (SPSS Inc., Chicago, IL, USA) was used for statistical analysis. All statistical analyses were two-sided, and a P-value<0.05 was considered statistically significant.

Categorical variables, expressed as numbers and frequencies, were compared using the χ^2^ test or Fisher exact test as appropriate. Continuous variables, presented as means ± SD, were compared using t-test or One-Way ANOVA. Univariate analysis was used to test for differences in demographic features and for all outcomes of this study among the three centers. Time of tracheal extubation was compared using the Kaplan-Meier survival method.

## Results

**Fig 1:**
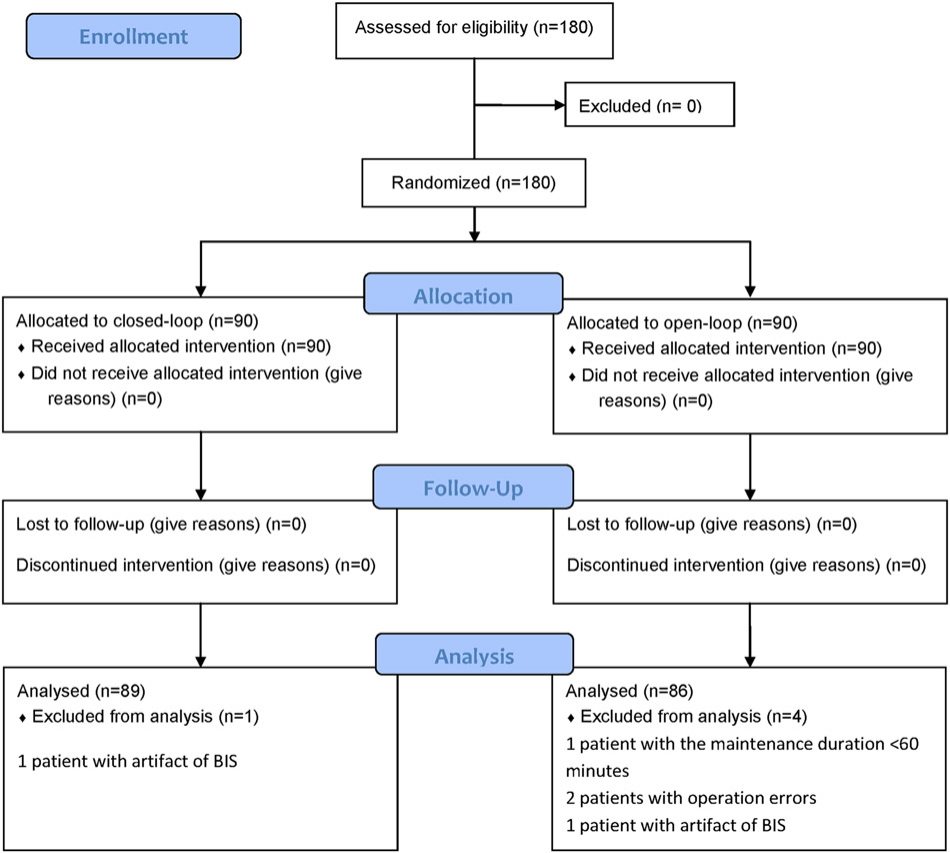
Patients’ flowchart. Of the 180 included patients, 1 and 3 patients were excluded from the closed-loop and opened-loop group, respectively, due to artifact of BIS, too short maintenance duration, or operation error. Thus, 89 and 86 patients were available for analysis in the two groups, respectively.

From 2012-12-13 to 2013-6-13 we had recruited 180 patients into the trial. Of the 180 included patients, 1 and 3 patients were excluded from the closed-loop group and opened-loop group, respectively, due to artifact of BIS, too short maintenance duration, or operation error (Fig 1). Thus, 89 and 86 patients were available for analysis in the two groups, respectively.

No significant site differences in the demographic variables were observed. There was no significant site-related difference in none of the outcome variables. While comparing preoperative co-morbidities, no significant differences in hypertension, diabetes, or coronary heart diseases were observed between the two groups. The operation types in the two groups included thoracic, hepatobiliary, gastrointestinal, urologic, gynecologic, and otorhinolaryngologic surgeries (Table 1).

**Table 1:** Medical and demographic characteristics of patients in the closed-loop group and the opened-loop group.

		Closed-loop (n = 89)	Opened-loop (n = 86)	P
Gender	Male (%)	31(35)	32(37)	0.744
Age	(years)	50.4±9.5	50.6±11.1	0.933
Height	(cm)	163.9±7.1	163.1±6.8	0.456
Weight	(kg)	63.7±9.5	62.7±9.4	0.491
BMI	(kg/m^2^)	23.7±3.3	23.6±3.0	0.752
ASA classification	I	40(45)	42(49)	
	II	49(55)	44(51)	0.607
Complications	Hypertension	17	15	
	Diabetes	7	3	
	Coronary heart disease	2	0	0.311
Operation type	Head and neck surgery	4(4)	6(7)	
	Chest wall and breast surgery	5(6)	2(2)	
	Thoracic surgery	1(1)	1(1)	
	Hepatobiliary surgery	14(16)	15(17)	
	Gastrointestinal surgery	29(33)	28(33)	
	Urologic surgery	7(8)	9(10)	
	Gynecologic surgery	29(33)	25(29)	0.897

Data are presented as mean ± SD; or number (%).

Closed-loop = automated control of propofol infusion group guided by the bispectral index; Opened-loop = manual control infusion group guided by the bispectral index; BMI = Body Mass Index.

Patients’ characteristics were also similar in the induction phase between the two groups, and no significant differences were observed between the two groups when comparing the induction target concentrations of propofol and remifentanil, as well as induction time (Table 2).

**Table 2:** Comparison of anesthetic procedures between the two groups during the induction phase.

		Closed-loop (n = 89)	Opened-loop (n = 86)	P
Midazolam	(mg)	1.76±0.43	1.77±0.41	0.887
Target concentration				
Propofol	(ug/ml)	2.88±0.29	2.86±0.34	0.569
Remifentanil	(ng/ml)	4.10±0.52	4.13±0.55	0.698
Induction time	(second)	201±163	240±216	0.174
SR	n (%)	0 (0)	0 (0)	1.000
Vasoactive drugs	n (%)	13(15)	16(19)	0.477

Data are presented as means± SD; or number (%).

Closed-loop = automated control of propofol infusion group guided by the bispectral index; Opened-loop = manual control infusion group guided by the bispectral index; The induction time = the time from the infusion of propofol and remifentanil to a BIS maintained at <60 for 30 seconds; SR = burst suppression ratio was calculated as SR>10% lasting at least one minute.

The mean dose of propofol was similar between the two groups during the maintenance phase (P>0.05). But the mean target concentration of propofol in the closed-loop group was lower than that in the opened-loop group. To maintain the BIS value in an adequate range during anesthesia, the frequency of propofol regulation in the closed-loop group (31.55±9.46 times/hr) was observed higher than that in the opened-loop group (6.84±6.21 times/hr) (p = 0.000). The doses of remifentanil and rocuronium were similar in the two groups (Table 3).

**Table 3:** Comparison of anesthetic procedures between the two groups during the maintenance phase.

		Closed-loop (n = 89)	Opened-loop (n = 86)	P
Maintenance time	(min)	199.3±96.2	202.5±101.0	0.832
Propofol				
Mean dose	(mg/kg∙h)	5.28±1.32	5.52±1.29	0.230
Mean target concentration	(μg/ml)	2.32±0.58	2.56±0.57	0.006
Adjusted times	(/h)	31.55±9.46	6.84±6.21	0.000
Remifentanil				
Mean dose	(μg/kg∙h)	11.14±3.08	11.05±3.30	0.848
Mean target concentration	(ng/ml)	5.01±1.25	4.87±1.22	0.465
Adjusted times	(/h)	2.62±2.06	3.61±2.68	0.007
Rocuronium				
Mean dose	(mg/kg∙h)	0.59±0.18	0.58±0.18	0.770
Time of dose added	(/h)	2.80±0.90	2.84±1.16	0.771
Blood loss >500ml	n (%)	11(12)	15(17)	0.345
Average transfusion volume	(ml/kg∙h)	9.77±3.56	10.07±4.15	0.606
Blood pressure adjustment	n (%)	30(33)	34(40)	0.440
Tramadol	(mg/kg)	2.03±1.27	2.05±1.08	0.889
Time to tracheal extubation	(min)	8.9±4.0	9.2±4.0	0.579

Data are presented as mean ± SD; or number (%).

Closed-loop = automated control of propofol infusion group guided by the bispectral index; Opened-loop = manual control infusion group guided by the bispectral index.

**Fig 2:**
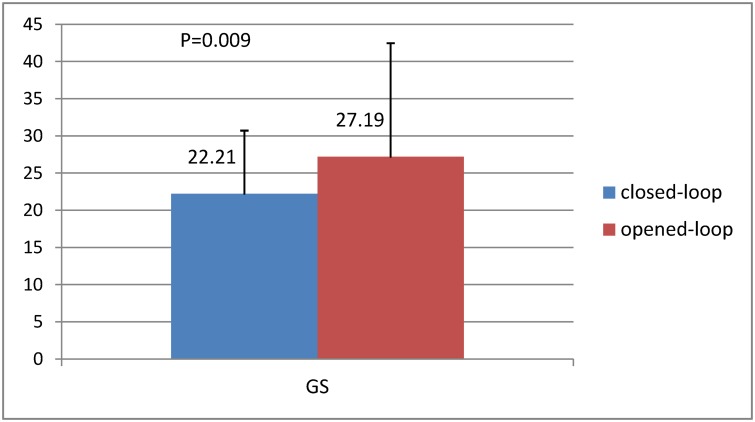
Comparison of the Global score (GS) between the two groups. Global score (GS) could reflect the overall performances of the closed-loop infusion system, including the fluctuation of BIS, the proportion of time of adequate anesthesia (BIS between 40 and 60), median absolute performance error (MDAPE), and Wobble. The mean GS were 22.21±8.50 and 27.19±15.26 in the closed-loop and opened-loop groups during the maintenance phase, respectively (p = 0.009).

**Fig 3:**
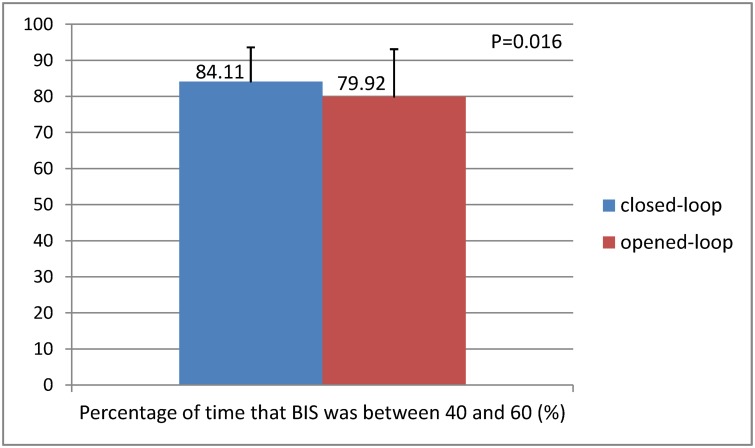
Comparison of the percentage of time that BIS was between 40 and 60 between two groups. The mean proportion of time that BIS was between 40 and 60 were 84.11±9.50% and 79.92±13.17% in the closed-loop group and opened-loop group, respectively (p = 0.016).

**Fig 4:**
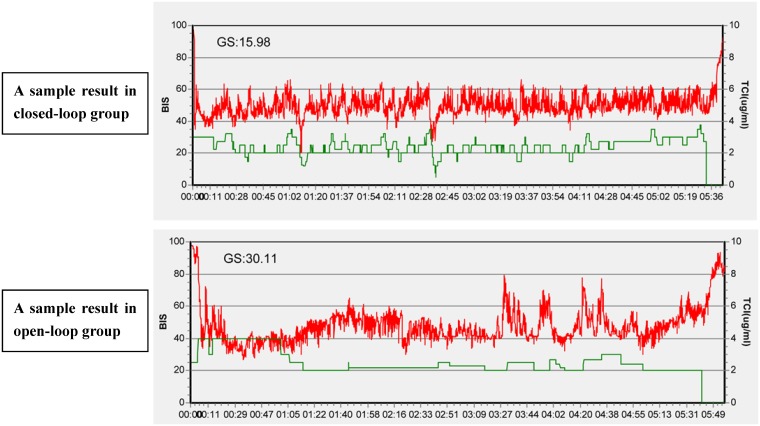
Two samples of the result of the trends of BIS and calculated target concentrations of propofol during anesthesia from the two groups. Two samples of the result of the trends of BIS (red lines) and calculated target concentrations of propofol (green lines) during the induction phase and maintenance phase in the two groups. The above was the closed-loop group (GS = 15.98) and the below was opened-loop group (GS = 30.11).

The mean GS were 22.21±8.50 and 27.19±15.26 in the closed-loop and opened-loop groups during the maintenance phase, respectively (p = 0.009) (Table 4 and Fig 2). With regard to the proportion of time that BIS was between 40 and 60, the higher proportions were observed in the closed-loop (84.11±9.50%), while the lower was found in the opened-loop group (79.92±13.17%) (p = 0.016) (Table 4 and Fig 3). Fig 4 is a sample of the result of the trends of BIS and calculated target concentrations of propofol in the two groups. (The original files are available as supporting information; see S1 BIS Data Report and S2 BIS Data Report.) PE, MDPE, and MDAPE were significantly lower in the closed-loop group compared with the opened-loop group. However, the Wobble scores were similar between the two groups. Over-anesthetization events were fewer in the closed-loop group compared with the opened-loop group, while only very few cases were found with insufficient anesthetization in the two groups. No patient was found with a SR>10% for at least 1 minutes in the present study.

**Table 4:** Effectiveness of the closed-loop control system.

	Closed-loop (n = 89)	Opened-loop (n = 86)	P
BIS<40 (%)	11.68±9.22	14.53±13.44	0.106
40<BIS<60 (%)	84.11±9.50	79.92±13.17	0.016
BIS>60 (%)	4.21±4.43	5.55±7.36	0.147
SR	0	0	1.000
Mean BIS	47.55±2.61	47.57±3.99	0.966
PE	-5.28±5.28	-5.23±7.49	0.963
MDPE	-5.28±5.44	-4.47±8.78	0.467
MDAPE	10.14±3.05	11.68±4.11	0.006
Wobble	7.98±2.20	8.23±2.42	0.483
GS	22.21±8.50	27.19±15.26	0.009

Data are presented as mean ± SD.

Closed-loop = automated control of propofol infusion group guided by the bispectral index; Opened-loop = manual control infusion group guided by the bispectral index; BIS<40(%) = percentage of time in which the BIS value was less than a value of 40; 40<BIS<60(%) = percentage of time in which the BIS value was between 40 and 60 during the maintenance; BIS>60(%) = percentage of time in which the BIS value was greater than a value of 60; SR = burst suppression ratio was calculated as SR>10% lasting at least one minute; PE = Performance error was defined as the difference between the actual value and the set value of BIS; MDPE = Median performance error; MDAPE = Median absolute performance error; Wobble = the intraindividual variability in PE; GS = Global score of BIS.

Times to tracheal extubation (from the end of the infusion of propofol and remifentanil to the time of endotracheal tube removal) were 8.9±4.0 and 9.2±4.0 minutes in the closed-loop and opened-loop groups, respectively (p = 0.579) (Table 3).

## Discussion

In the present study, the results of GS, MDAPE, and the proportion of time of BIS between 40 and 60 demonstrated that the closed-loop infusion system controlled BIS slightly better than the opened-loop control group did.

Previous studies have demonstrated that BIS could be used as a target for closed-loop infusion of propofol [8–11,14,15]. The BIS values could be used to help regulating the infusion of propofol in closed-loop infusion, which could effectively avoid the limitations of the pharmacokinetics patterns of TCI.

In the present study, the system was connected to a BIS monitor. It automatically collected a BIS value from the monitor every 5 seconds, and calculated the mean BIS value every 3 minutes. Then the mean BIS values were classified into different levels, with each level corresponding to a target concentration of propofol. The system could then automatically regulate the target concentration of propofol according to the BIS value level. In some cases, the BIS value could not be maintained between 45 and 55 for a certain time period (mostly 3 minutes), and the system regulated the target concentration of propofol of each BIS level until the BIS value was between 45 and 55. The mean BIS value was calculated every 3 minutes to avoid a too frequent regulation of the infusion dose of propofol caused by possible disturbances of the BIS. The regulation time was also set at 3 minutes for the present study, which was also selected according to previous experiences that a too long regulation time could increase the risk of BIS fluctuation. The system also allowed maintaining the target concentration of the drugs according to the data collected automatically, even if some disturbances in the system or BIS occurred (S1 Appendix).

Although using this system, either automatical or manual administration of propofol, could achieve appropriate anesthesia depth during surgeries with general anesthesia, the proportion of time of BIS between 40 and 60, which was regarded as adequate anesthesia, was longer in the closed-loop group than in the opened-loop group, suggesting that the regulation of propofol was more precise in the closed-loop group. The regulation of propofol could be about 31.16±9.95 times/hour after the modification to the protocol, which is not possible to perform manually. There was clinical significance in reducing the workload of anesthesiologists. Then it would release some efforts and time for the anesthesiologist to be even more attentive to the surgical procedure, or for that matter to other organ systems that should be monitored as well.

Endotracheal tube removal time is one of the parameters of recovery quality of the patients. The findings of the present study showed that the endotracheal tube removal time was about 0.3 minutes shorter in the closed-loop than in the opened-loop group. However, the difference was not statistically significant, suggesting that the recovery quality was similar in the two groups.

The findings of the present study also suggest that there were some limitations to this closed-loop infusion system. The first limitation was that we chose the plasma concentration directed Marsh model and 3 minutes as the time span for the moving BIS-average in order to reduce the impact on the hemodynamics. Thus the adjustment may be slow. The second limitation was the use of remifentanil. Previous studies have demonstrated that pain could affect BIS, and that surgery could increase BIS in cases of insufficient analgesia [17,18]. However, no parameter has been acknowledged that could effectively evaluate pain level; thus, hemodynamic changes have been used by the clinicians to estimate pain level. This experience-based evaluation method for choosing the infusion target concentration of remifentanil could affect the stability of BIS in the closed-loop infusion system, especially when the system was operated by different clinicians. However, no significant difference in the use of remifentanil between the two groups was observed, suggesting that this limitation was basically avoided in the present study. The third limitation was that only patients aged among 18 and 65 years were included, while elderly patients (>65 years) or critically ill patients were not included, which could introduce a selection bias and should be avoided in future studies.

Another limitation which must be discussed was the hysteresis of adjusting infusion of propofol by the closed-loop system. This limitation was based on the working principle of this closed-loop system. But before conceivable stimulation occurred, experienced anesthesiologist could always increase the target concentration of remifentanil to avoid hemodynamics disorder. Closed-loop system was only a valuable tool to assist the anesthesiologist in controlling anesthetic infusion and reduce the workload. Anesthesiologist must be present all the time to overlook the system and will always hold the ultimate responsibility for patient safety.

## Conclusion

The CONCERT-CL closed-loop infusion system could automatically regulate the TCI of propofol under the guide of BIS, maintain the BIS value in an adequate range and reduce the workload of anesthesiologists better than open-loop system.

## Supporting Information

S1 Appendix The introduction of the closed-loop controlled infusion system (CONCERT-CL). (DOC)

S1 CONSORT Checklist (DOC)

S1 Dataset (XLS)

S1 Protocol CONCERT CL(Chinese) (DOC)

S1 Protocol CONCERT CL(English) (DOC)

S1 BIS Data Report (PDF)

S2 BIS Data Report (PDF)
