# Supplementary material for: Closed-Loop Control Better than Open-Loop Control of Profofol TCI Guided by BIS: A Randomized, Controlled, Multicenter Clinical Trial to Evaluate the CONCERT-CL Closed-Loop System
Source: PLoS One. 2015 Apr 17;10(4):e0123862. doi: 10.1371/journal.pone.0123862 (PMC4401751; doi:10.1371/journal.pone.0123862)
Supplement: S1 Appendix — (DOC) [file pone.0123862.s001.doc]

**The appendix:**

The closed-loop controlled infusion system (CONCERT-CL) designed by VERYARK Technology Co., Ltd. (Guangxi, China) is composed of three infusion pathways (A, B, and C) controlled by a core processing system. Pathway A mainly regulates the TCI of propofol by monitoring the brain electrical activity(using BIS) during the induction and maintenance of general anesthesia. The pathway B mainly regulates the TCI of remifentanil. The pathway C mainly regulates the closed-loop infusion of muscular relaxants under the monitoring of neuromuscular blockade. The system may be switched to a manual regulation mode.

In the pathway A, the system is connected to a BIS monitor (AspectA-2000XP BIS), and automatically collects BIS values every 5 seconds, and the mean BIS values within every 3 minutes are calculated. Then, the BIS values are used to automatically regulate the target concentration of propofol. In some cases, the BIS value cannot be maintained between 45 and 55 during a given time period (mostly 3 minutes), and the system will then regulate the target concentration of propofol of each BIS level until the BIS value is between 45 and 55.

In the present study, the system was connected to a BIS monitor. It automatically collected a BIS value from the patients every 5 seconds, and calculated the mean BIS value every 3 minutes. Then the mean BIS values were classified into different levels, with each level corresponding to a target concentration of propofol. The system could then automatically regulate the target concentration of propofol according to the BIS value level. In some cases, the BIS value could not be maintained between 45 and 55 for a certain time period (mostly 3 minutes), and the system regulated the target concentration of propofol of each BIS level until the BIS value was between 45 and 55. The mean BIS value was calculated every 3 minutes to avoid a too frequent regulation of the infusion dose of propofol caused by possible disturbances of the BIS. The regulation time was also set at 3 minutes for the present study, which was also selected according to previous experiences that a too long regulation time could increase the risk of BIS fluctuation. The system also allowed maintaining the target concentration of the drugs according to the data collected automatically, even if some disturbances in the system or BIS occurred.
