# Supplementary material for: Closed-Loop Control Better than Open-Loop Control of Profofol TCI Guided by BIS: A Randomized, Controlled, Multicenter Clinical Trial to Evaluate the CONCERT-CL Closed-Loop System
Source: PLoS One. 2015 Apr 17;10(4):e0123862. doi: 10.1371/journal.pone.0123862 (PMC4401751; doi:10.1371/journal.pone.0123862)
Supplement: S1 Protocol CONCERT CL(Chinese) — (DOC) [file pone.0123862.s004.doc]

**医疗器械临床试验方案**

产品名称：

全凭静脉三通监控自动注射系统

型号规格：

CONCERT-CL

实施者：

广西威利方舟科技有限公司

承担临床试验的医疗机构：

1.首都医科大学附属北京朝阳医院

2.复旦大学附属上海中山医院

3.华中科技大学附属武汉协和医院

临床试验负责人：（签字）岳云

| 临床试验的背景：  随着丙泊酚、雷米芬太尼等速效、短效的全身麻醉药物的出现，全凭静脉麻醉（TIVA）作为近十年来一种新兴的全身麻醉方式，以其起效快、维持平稳、恢复迅速、苏醒质量高、没有麻醉气体的污染问题等特点，深得广大麻醉医师的推崇。靶控输注（TCI）的出现使得TIVA的药物应用更加合理，更加符合药代动力学和药效动力学模型，因此TCI也被越来越多的麻醉医师所接受，但是TCI本身的先天缺陷是其应用的药代动力学模型不可能适用于所有患者，不同患者的个体差异决定了TCI存在误差。近年来国内外对于全麻药物的闭环（Closed-Loop）输注研究开始增加，应用闭环方法输注全麻药物可以弥补TCI的缺陷，最大程度的解决患者的个体差异问题，达到合理给药、适量用药。  脑电双频指数（BIS）是一种脑电图（EEG）监测方法，已经被证明可以指导全麻中镇静药物水平的调节，并广泛应用于有关丙泊酚输注的对比研究中。因此BIS可以作为丙泊酚闭环输注的靶目标。  广西威利方舟科技有限公司（简称威利方舟）前些年生产的闭环肌松注射系统（CLMRIS）是应用肌松监测（TOF）结果进行反馈控制肌松剂的输注，达到肌松剂的闭环输注，取得了良好地临床效果。 |
| --- |
| 产品的机理、特点与试验范围：  威利方舟生产的全凭静脉三通监控自动注射系统，是在原有成熟的双通道靶控注射泵基础上结合闭环肌松注射系统，开发出的新一代注射系统。此款产品分为三个输注通道，A通道的主要功能是在BIS等脑电监测结果（系统通过数据线与BIS等监测仪相连并采样，每5秒采集一个BIS值）的指导下闭环输注丙泊酚；B通道的主要功能是雷米芬太尼的TCI；C通道的主要功能是在TOF监测结果的指导下闭环输注肌松剂，即先前的闭环肌松注射系统（CLMRIS）改进版。  因为B和C通道的应用已经通过了临床验证，因此此次试验主要是针对A通道的临床验证。  A通道的作用机理：将患者的BIS值（实际为3分钟均值）分为不同的区间，每个区间对应一个丙泊酚靶浓度，可列为一个表格，当BIS值在不同的区间时，系统自动调节相应的靶浓度。若在一定时间（2分钟）内按照当前表格无法将BIS值控制在50左右（45~55），系统会根据BIS值大于55或小于45而调整表格，将不同区间对应的靶浓度上调或下调，直到BIS值被控制在45~55之间。  此产品经国家食品药品监督管理局批准，由公司委托首都医科大学附属北京朝阳医院负责临床验证。 |
| 临床试验的项目内容和目的：  研究名称：应用全凭静脉三通监控自动注射系统CONCERT-CL输注丙泊酚的优势性和安全性的临床验证。试验目的：验证此款三通道闭环注射系统在全麻手术患者术中闭环输注丙泊酚能够自动达到合适的镇静水平，优于手工输注方法，同时验证其在临床应用上是安全的。 |
| 总体设计（包括成功和失败的可能性分析）：  本研究将患者分为两组：闭环（闭环组）或人工（人工组）TCI丙泊酚组。应用一个随机数字表对患者进行随机分组。  患者入手术室后，开放静脉通路，进行常规监测（血压、心率、脉搏氧饱和度）和在拇内收肌处进行肌松监测，并在患者额头放置BIS电极，进行BIS监测。常规给予咪唑安定（力月西，恩华药业）1~2mg。  两个组的诱导阶段定义为从丙泊酚（得普利麻，阿斯利康）和雷米芬太尼（瑞捷，宜昌人福）输注开始到BIS低于60达30秒为止，维持阶段为从该时点到丙泊酚和雷米芬太尼输注结束。  诱导阶段，研究者根据临床经验选择用于诱导的丙泊酚(2-4ug/ml)和雷米芬太尼(4-8ng/ml)的初始靶浓度。维持阶段，在人工组中手动调节丙泊酚靶浓度维持BIS在50左右（40~60），在闭环组中，由系统自动调节丙泊酚靶浓度；两组的雷米芬太尼应用B通道TCI人工输注，靶浓度范围在2~8ng/ml；肌松剂使用罗库溴铵（爱可松，默沙东），应用C通道进行闭环输注，反馈条件统一设为计数2，其他参数由研究者根据临床经验设定。在手术过程中，如果有必要，研究者可以停止闭环输注改为手工调节。  除了输注研究药物外，研究者根据现有的标准进行麻醉管理。对血流动力学异常的处理没有特殊建议。术中不使用吸入麻醉剂。预计手术结束前30分钟停止肌松剂的输注。预计手术结束前20分钟，给予曲马多和止吐药。  两组患者在手术结束时同时停止丙泊酚和雷米芬太尼的输注，常规给予肌松拮抗剂（阿托品1mg+新斯的明2mg）。满足下述所有条件时拔除气管导管或喉罩：患者有反应、能配合；自主呼吸恢复，SpO_2_>95%；T4/T1>90%；无血流动力学紊乱。  主要观察指标是总体分数（GS），它反映了包括充分麻醉（BIS在40~60之间）所占时间比例以及由执行误差绝对中位数（MDAPE）和摆动（Wobble）决定的BIS波动在内的闭环系统的整体性能。通过以下公式计算系统性能。  ·执行误差（PE），是实际值与设定值之差：  PE_ij_=[(BIS_测量ij_-BIS_设定_)/BIS_设定_]×100  ·偏差或执行误差中位数（MDPE）：  MDPE_i_=中位数[PE_ij_，j=1，…，N_i_]  ·不准确度或执行误差绝对中位数（MDAPE）：  MDAPE_i_=中位数[｜PE_ij_｜，j=1，…，N_i_]  ·摆动，反映了PE的个体差异：  Wobble_i_=[｜PE_ij_-MDPE_i_｜，j=1，…，N_i_]  i代表研究对象的编号，j代表观察期的第j次测量结果，N代表观察期间的总观察数。  总体分数（GS）：  GS=(MDAPE+Wobble)/% (BIS值在40~60时)  低MDAPE和Wobble同时BIS值在40~60所占时间比例高，并由此得到低的GS说明该闭环系统的性能好。  次要指标包括充分麻醉（40<BIS<60）、麻醉过深（BIS<40）和麻醉过浅（BIS>60）所占的时间比，爆发抑制比（SR）即SR>10%持续至少1分钟，以及PE、MDPE、MDAPE、Wobble。  次要指标还包括：丙泊酚、雷米芬太尼和罗库溴铵的药物用量、罗库溴铵闭环给药的效果（是否需要手动加药、临床满意度等）、体动次数、气管拔管时间（即从停止输注丙泊酚和雷米芬太尼到拔管的时间）等。  本试验中，PE、MDPE、MDAPE、Wobble和GS，以及BIS值不同条件时间占比等统计数据由威利方舟公司提供的数据采集软件获得。每完成一例病例会在注射系统中存留一个数据文件，通过数据线连接电脑，通过数据采集软件导入电脑中进行分析，可以查看BIS趋势图表、药物靶浓度趋势图表、肌松监测趋势图表等，并可以自动得出试验需要的统计数据，生成统计报告并打印。  试验结束后对以下方面进行评价：两组最终GS有无差异，两组间的用药量有无差异，研究者对系统的整体评分。  同时记录相关不良反应情况，如果出现需说明其与试验系统有无相关性。 |
| 临床评价标准：  1.优势评价：若GS结果闭环组<人工组，且在统计学上有显著差异，说明闭环输注优于手工输注。  2.安全性评价：若术中未出现与试验系统相关的不良反应，则说明其在临床应用上是安全的。 |
| 每病种临床试验例数及其确定理由：  本试验预期应用闭环系统得到的GS相对于人工输注得到的GS能有超过50%的改善率。要得到一个双侧α为0.01，检验效力为95%，预计需要144例患者（每组72例）。鉴于某些患者可能因各种原因退出研究，我们计划选择180例患者（每组90例），分3个中心进行研究，本中心计划完成60例（每组30例）。 |
| 选择对象范围（包括必要时对照组的选择），选择对象数量及选择理由：  1．择期在全麻下手术的患者；  2．预计手术时间超过120分钟；  3．年龄为18-65岁；  4. 体重指数BMI不超过正常范围20%；  5．ASA分级I-II级；  6．手术体位适合进行BIS监测及肌松监测；  7．获得知情同意。 |
| 临床性能的评价方法和统计处理方法：  1．统计分析将采用SPSS 11.0统计分析软件进行计算。统计检验采用双侧检验，P<0.05将被认为差异具有统计学意义。  2.试验中的计数资料采用χ^2^检验或Fisher精确检验进行比较。计量资料采用均数±标准差表示，采用t检验或Mann-Whitney U检验进行比较。系列测量数据用重复测量方差分析进行比较，多重比较使用非参检验。拔管时间用Kaplan-Meier生存分析进行比较，然后用log-rank进行检验。  3．安全性分析：两组不良反应发生率比较采用χ^2^检验，并描述其与本试验相关性。  4．性能评价：对统计结果采用描述性分析，进行闭环系统的优势性评价、安全性评价及整体评分。 |
