# Supplementary material for: Closed-Loop Control Better than Open-Loop Control of Profofol TCI Guided by BIS: A Randomized, Controlled, Multicenter Clinical Trial to Evaluate the CONCERT-CL Closed-Loop System
Source: PLoS One. 2015 Apr 17;10(4):e0123862. doi: 10.1371/journal.pone.0123862 (PMC4401751; doi:10.1371/journal.pone.0123862)
Supplement: S1 Protocol CONCERT CL(English) — (DOC) [file pone.0123862.s005.doc]

**The Trial Study Protocol of Medical Device**

**Name of the Device**：

The CONCERT-CL closed-loop infusion system

**Model Number**：

CONCERT-CL

**Name of Manufactory**：

VERYARK Technology Co., Ltd. (Guangxi, China)

**Medical Institution of Clinical Trial**：

1. Department of Anesthesiology, Beijing Chaoyang Hospital, Capital Medical Univesity, Beijing, People’s Republic of China;

2. Department of Anesthesiology, Zhongshan Hospital, Fudan University, Shanghai, People’s Republic of China;

3. Department of Anesthesiology, Union Hospital, Tongji Medical College, Huazhong University of Science and Technology, Wuhan, People’s Republic of China.

**Responsible Person**:

Yun Yue

| **Background**：  With the development of fast- but short-acting anesthetics, such as propofol and remifentanil, total intravenous anesthesia (TIVA) has been widely accepted because it is fast-acting and stable, and allows for a rapid recovery. Advances in target controlled infusion (TCI) further improved TIVA to better fit the pharmacokinetics and pharmacodynamics of these drugs. However, individual differences among patients impair the application of the pharmacokinetic model of TCI in some patients. For this reason, closed-loop controlled infusion has become a hot topic in recent years. Closed-loop controlled infusion of anesthetics allows avoiding the limitations of TCI by compensating the disturbances caused by individual differences, and thus helps to achieve a rational use of anesthetics.  Bispectral index (BIS), a FDA-approved index that have been widely used to monitor the depth of anesthesia, has been used in several comparative studies involving the infusion of propofol, and could help regulating the infusion of anesthetics during general anesthesia. BIS is regarded as the best index for monitoring closed-loop controlled infusion of propofol. |
| --- |
| **Introduction of the Device**：  The closed-loop controlled infusion system (CONCERT-CL) designed by VERYARK Technology Co., Ltd. (Guangxi, China) is composed of three infusion pathways (A, B, and C) controlled by a core processing system. Pathway A mainly regulates the TCI of propofol by monitoring the brain electrical activity (using BIS) during the induction and maintenance of general anesthesia. The pathway B mainly regulates the TCI of remifentanil. The pathway C mainly regulates the closed-loop infusion of muscular relaxants under the monitoring of neuromuscular blockade. The system may be switched to a manual regulation mode.  In the pathway A, the system is connected to a BIS monitor (AspectA-2000XP BIS), and automatically collects BIS values every 5 seconds, and the mean BIS values within every 3 minutes are calculated. Then, the BIS values are used to automatically regulate the target concentration of propofol. In some cases, the BIS value cannot be maintained between 45 and 55 during a given time period (mostly 3 minutes), and the system will then regulate the target concentration of propofol of each BIS level until the BIS value is between 45 and 55. |
| **Aim of the Trial**：  The aim of the trial is to investigate whether the CONCERT-CL system could be better to stabilize the BIS and maintain the BIS value between 40 and 60 by comparing the effects of BIS-guided regulated closed-loop TCI of propofol and manually regulated TCI of propofol. |
| **Clinical Trial Design**：  The patients are randomly assigned to the closed-loop or opened-loop group using a random number table. Group assignment will be enclosed in opaque envelopes before the operation.  Blood pressure, electrocardiogram, and pulse oximetry are monitored. Neuromuscular blockade at the abductor pollicis muscle is monitored using the neuromuscular blockade monitoring system provided by VERTARK Technology Co., Ltd. (Guangxi, China), while BIS is monitored using an A-2000XP BIS (Aspect Medical systems, Dublin, Ireland). Midazolam 1~2mg will be administered as a premedication.  The induction phase is defined as from the infusion of propofol (Diprivan, AstraZeneca, London, UK) and remifentanil to a BIS maintained at <60 for 30 seconds. The maintenance phase is defined as from the end of the induction to the end of the infusion of propofol and remifentanil  The initial target concentrations of propofol in the plasma (2 to 4 ug/ml) and remifentanil (4 to 8 ng/ml) in the induction phase are selected by the anesthesiologists according to their clinical experience. In the maintenance phase, the target concentration of propofol is adjusted manually to maintain the BIS at about 50 (40 to 60) in the opened-loop group, while the target concentration of propofol in the closed-loop group is adjusted automatically by the system. The TCI of remifentanil is used in both groups, and the target concentration (2 to 8 ng/ml) is based on clinical judgment of the anesthesiologists.  The closed-loop infusion of rocuronium (Esmeron, Merck Forsst, Montreal, Canada) will be used after the induction phase. The induction dose of rocuronium is 0.6 mg/kg, and then the feedback parameter is at reappearance of second twitch (the count 2) for maintenance infusion of rocuronium. Endotracheal intubation or laryngeal mask insertion is performed when TOFr=0. The anesthesiologists can administer the drugs manually or switch the closed-loop infusion to manual infusion of the drugs during the operation, if needed.  All aspects of anesthesia managements except for the drug infusion are performed by the anesthesiologists according to the currently used guidelines. No specific treatments are used for patients with abnormal hemodynamics. No inhalation anesthetic is used. The infusion of the muscle relaxants will be stopped at about 30 minutes before the end of the operation, and 100 to 200 mg of tramadol will be administered at about 20 minutes before the end of the operation.  The infusion of propofol and remifentanil will be stopped at the same time after the operation in both groups. Then, muscle-relaxant antagonists (1 mg of atropine and 2 mg of neostigmine) are administered. The endotracheal tube or laryngeal mask will be removed when the patients reached consciousness, can respond to the clinicians, have a restored autonomous respiration, SpO_2_>95%, TOFr>90%, and are without hemodynamic disturbance.  Global score (GS) can reflect the overall performances of the closed-loop infusion system, including the fluctuation of BIS, the proportion of time of adequate anesthesia (BIS between 40 and 60), median absolute performance error (MDAPE), and Wobble [13]. Therefore, GS is selected as the primary outcome. The parameters are calculated as follows:  Performance error (PE) is defined as the difference between the actual value and the set value:  PEij=[(BISactualij-BISset)/BISset]×100.  Median performance error (MDPE):  MDPEi=Median [PEij，j=1，…, Ni]  Median absolute performance error (MDAPE):  MDAPEi=Median [｜PEij｜，j=1, …，Ni]  Wobble reflects the intraindividual variability in PE:  Wobblei=[｜PEij-MDPEi｜，j=1, …，Ni]  i=subject number; j=jth (one) measurement of observation period; N=total number of measurements during the observation period.  Global score (GS) is calculated using the formula:  GS=(MDAPE+Wobble)/% of time BIS between 40 and 60  A lower GS, meaning lower MDAPE, lower Wobble, and higher proportion of time of BIS between 40 and 60, represent better performances of the closed-loop infusion system.  The secondary outcomes include the percentage of adequate anesthesia (BIS between 40 and 60), overshoot (BIS<40) and undershoot (BIS>60) periods, occurrence of suppression ratio (SR) defined as SR>10% lasting at least one minute, and parameters (PE, MDPE, MDAPE, Wobble). And the adjustment times per hour for control of adequate anesthesia (BIS between 40 and 60).  The secondary outcomes also include doses of propofol, remifentanil and rocuronium, and the endotracheal tube removal time (from the end of the infusion of propofol and remifentanil to the removal of the endotracheal tube).  PE, MDPE, MDAPE, Wobble, GS, and the proportion of the time of BIS are collected automatically by the data-collecting software provided by VERYARK Technology Co., Ltd. The trends of BIS, target concentration of the drugs, and neuromuscular blockade can be displayed. |
| **The Number of Each Group and Reason**  In this trial we expect an improvement of >50% of GS using the closed-loop system. We thus estimate that 144 patients (72 per group) will provide a 95% power for a 2-sided α error of 0.01. We plan to recruit 180 patients (60 per hospital) under the assumption that some will be excluded for various reasons. |
| **Subject Inclusion Criteria**  1．General anesthesia;  2．Expected operation time>120 minutes;  3．Age ranged from18 to 65 years;  4. BMI within 20% of the standard range;  5．ASA I-II;  6．Surgical position suitable for BIS and TOF monitor;  7．Obtain informed consent. |
| Subject Exclusion Criteria   | 1. Spirit system disease; | | --- | | 1. Central and peripheral nervous system disorders; | | 1. Neuromuscular system disorders; | | 1. Cerebral nerve surgery; | | 1. Pacemakers; | | 1. Surgical position not suitable for BIS and TOF monitor; | | 1. Hemodilution or hypothermia. | |
| **Statistical analysis**  SPSS 19.0 (SPSS Inc., Chicago, IL, USA) is used for statistical analysis. All statistical analyses are two-sided, and a P-value<0.05 is considered statistically significant.  Categorical variables, expressed as numbers and frequencies, are compared using the χ2 test or the Fisher exact test, as appropriate. Continuous variables, presented as means ±SD, are compared using t-test or ANOVA. Comparison of serial measurements is performed with repeated-measures ANOVA, and post hoc analyses are performed with nonparametric tests. Time of tracheal extubation is compared using the Kaplan-Meier survival method. |
