# Supplementary material for: Closed-Loop Control Better than Open-Loop Control of Profofol TCI Guided by BIS: A Randomized, Controlled, Multicenter Clinical Trial to Evaluate the CONCERT-CL Closed-Loop System
Source: PLoS One. 2015 Apr 17;10(4):e0123862. doi: 10.1371/journal.pone.0123862 (PMC4401751; doi:10.1371/journal.pone.0123862)
Supplement: S1 BIS Data Report — (PDF) [file pone.0123862.s006.pdf]

BIS闭环数据分析报告

注射信息：

日期: 2013/1/11	开始时间: 12:08:11
病例号: 000114	体重: 66 kg
年龄: 39	性别: 女

趋势图：

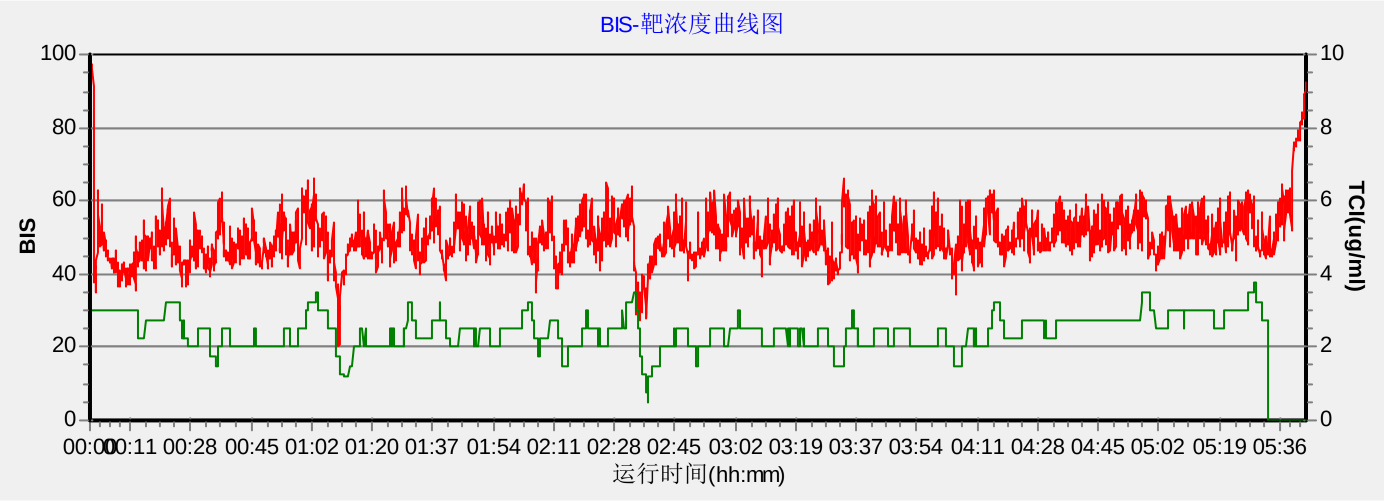

数据分析结果：

	诱导期数据	
A通道起始靶浓度:3.0ug/ml	B通道起始靶浓度:4.0ng/ml	
诱导时间:98秒	BIS<40(3min):0秒	BIS>60(3min):15秒
	维持阶段数据	
麻醉维持时间:331分钟		
区间百分比(40-60):91.4%	区间百分比(<40): 3.4%	区间百分比(>60): 5.2%
丙泊酚总量:1889.26 mg		
5.18 mg/kg/h	调整次数:166(30.02次/h)	平均靶浓度:2.4
瑞芬总量:4720.76 ug		
12.93 ug/kg/h	调整次数:9(1.63次/h)	平均靶浓度:5.8
BIS最大值:66.1	BIS最小值:20.2	BIS平均值:49.7
PE:-0.64		
MDPE:-1.80	MDAPE:7.40	Wobble:7.20
GS:15.98		

打印时间: 2014/4/5 2:07:48
