# Supplementary material for: Closed-Loop Control Better than Open-Loop Control of Profofol TCI Guided by BIS: A Randomized, Controlled, Multicenter Clinical Trial to Evaluate the CONCERT-CL Closed-Loop System
Source: PLoS One. 2015 Apr 17;10(4):e0123862. doi: 10.1371/journal.pone.0123862 (PMC4401751; doi:10.1371/journal.pone.0123862)
Supplement: S2 BIS Data Report — (PDF) [file pone.0123862.s007.pdf]

BIS闭环数据分析报告

注射信息：

日期: 2013/1/18	开始时间: 09:14:07
病例号: 000121	体重: 49 kg
年龄: 54	性别: 女

趋势图：

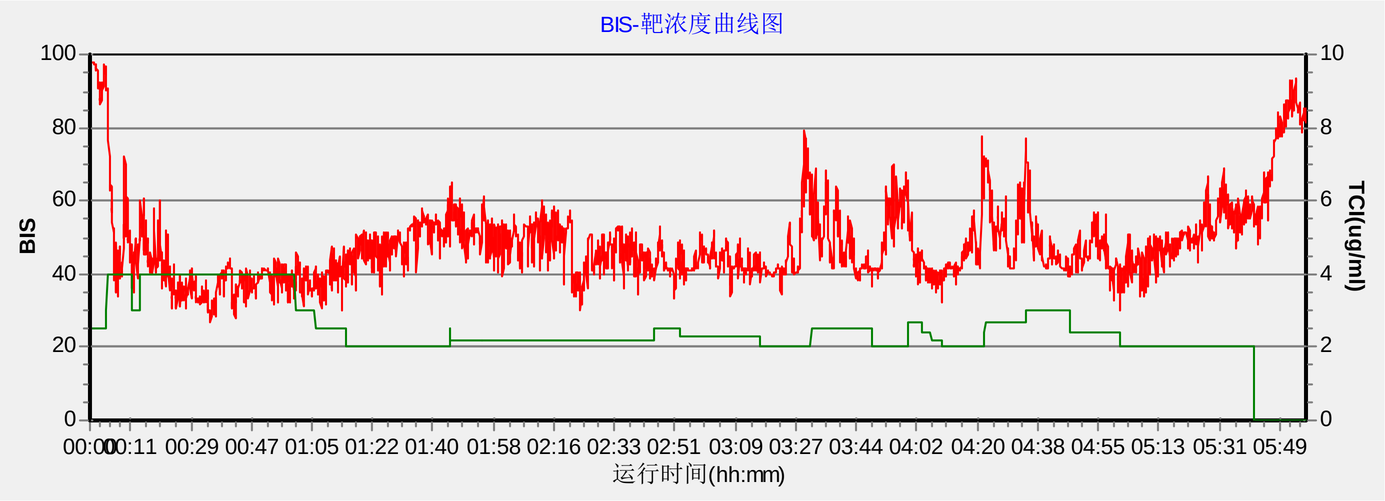

数据分析结果：

	诱导期数据	
A通道起始靶浓度:2.5ug/ml	B通道起始靶浓度:4.0ng/ml	
诱导时间:434秒	BIS<40(3min):55秒	BIS>60(3min):5秒
	维持阶段数据	
麻醉维持时间:334分钟		
区间百分比(40-60):77.0%	区间百分比(<40):18.1%	区间百分比(>60): 4.9%
丙泊酚总量:1357.19 mg		
4.97 mg/kg/h	调整次数:21(3.77次/h)	平均靶浓度:2.5
瑞芬总量:4582.66 ug		
16.79 ug/kg/h	调整次数:9(1.62次/h)	平均靶浓度:6.7
BIS最大值:79.1	BIS最小值:27.0	BIS平均值:46.2
PE:-7.65		
MDPE:-10.40	MDAPE:13.60	Wobble:9.60
GS:30.11		

打印时间: 2014/4/5 2:08:45
